# Suggestive evidence of a multi-cytokine resistin pathway in humans and its role on cardiovascular events in high-risk individuals

**DOI:** 10.1038/srep44337

**Published:** 2017-03-14

**Authors:** Claudia Menzaghi, Antonella Marucci, Alessandra Antonucci, Concetta De Bonis, Lorena Ortega Moreno, Lucia Salvemini, Massimiliano Copetti, Vincenzo Trischitta, Rosa Di Paola

**Affiliations:** 1 Research Unit of Diabetes and Endocrine Diseases, IRCCS “Casa Sollievo della Sofferenza”, San Giovanni Rotondo, Italy; 2 Unit of Biostatistics, IRCCS “Casa Sollievo della Sofferenza”, San Giovanni Rotondo, Italy; 3 Department of Experimental Medicine, Sapienza University of Rome, Italy

## Abstract

In cells and tissues resistin affects IL-1β, IL-6, IL-8, IL-12 and TNF-α expression, thus suggesting the existence of a multi-cytokine “resistin pathway”. We investigated whether such pathway does exist in humans and, if so, if it is associated with cardiovascular risk factors and with major adverse cardiovascular events (MACE). Serum cytokines were measured in 280 healthy subjects from the Gargano Study 2 (GS2) whose BMI, waist circumference, HOMA_IR_, triglycerides, HDL-cholesterol, systolic and diastolic blood pressure data were available and in 353 patients with type 2 diabetes and coronary artery disease from the Gargano Heart Study (GHS)-prospective design (follow-up 5.4 ± 2.5 years; 71 MACE). In GS2, cytokines mRNA levels in white blood cells were also measured. In GS2, resistin mRNA was correlated with all cytokines expression (all p < 0.001), but IL-12B. Consistently, serum resistin was correlated with all serum cytokines (all p < 0.001), but IL-12. Expression (eRPS) and serum (sRPS) resistin pathway scores (excluding IL-12) were each other correlated (p < 0.001) and both associated with cardiovascular risk factors (all p < 0.01). In GHS, sRPS was independently associated with MACE (HR = 1.44, 95% CI = 1.10–1.90). Our data indicate the existence of a resistin pathway, which is associated with cardiovascular risk factors and which strongly and independently predicts MACE.

Cardiovascular disease is the major cause of morbidity and mortality worldwide^1^. This deleterious role is exacerbated in patients with type 2 diabetes^2^, in which insulin resistance, obesity, hypertension, dyslipidemia and related treatments also contribute to cardiovascular risk. A better understanding of the pathogenic mechanisms shaping atherosclerotic processes is, therefore, urgently needed in order to tackle such heavy burden, affecting patients and their relatives.

Insulin resistance (IR) and chronic low-grade inflammation (LGI) have been recognized, among other components, as prime movers of atherosclerotic processes and, eventually, cardiovascular disease^3^,^4^. Several cytokines have been linked to both conditions^5^. Among these is resistin, whose circulating and/or tissue expression levels have been associated with atherosclerosis and related clinical phenotypes, including major adverse cardiovascular events (MACE)^6^,^7^,^8^,^9^,^10^,^11^,^12^,^13^,^14^,^15^,^16^,^17^,^18^,^19^,^20^,^21^.

Recently, it has been shown that resistin affects, in white blood cells, macrophages, hepatic stellate cells and adipose tissue the expression of other cytokines, including IL-1β, IL-6, IL-8, IL-12 and TNF-α^22^,^23^,^24^,^25^,^26^, so that in cellular and tissue models the existence of a multi-cytokine “resistin pathway” can be hypothesized.

Whether a resistin pathway does exist in humans and, if so, whether it does play a detrimental role on atherosclerosis and clinical outcomes related to IR and LGI is not known.

Aims of our study were as it follows: i) to explore in humans the plausibility of a resistin pathway by testing the correlations between resistin and those cytokines whose expression has been experimentally reported to be under the direct control of resistin itself, including IL-1β, IL-6, IL-8, IL-12 and TNF-α levels from both serum and blood cell mRNA of healthy individuals; ii) to create a multi-marker resistin pathway score, both from serum (sRPS) and from mRNA expression (eRPS) data and to test, in the same healthy individuals, the association with several cardiovascular risk factors (CRF) related to both IR and LGI; iii) to test prospectively the association between sRPS and MACE in high-risk patients. To address these aims, two cohorts, namely Gargano Study 2 (GS2), including healthy and with no ongoing treatments individuals and Gargano Heart Study (GHS)-prospective design, including subjects affected by type 2 diabetes and established coronary artery disease were investigated.

In our study design, GS2 has served the function to address the plausibility of a resistin pathway in healthy individuals and its correlation with traits known to affect IR- and LGI-related cardiovascular risk factors. Conversely, based on our positive findings in GS2, GHS-prospective design was used to investigate whether resistin pathway predicts MACE in high-risk individuals.

## Methods

### Study population

#### Subjects from the Gargano study 2 (GS2)

This sample comprised 280 unrelated healthy blood donors with a wide range of BMI belonging to the GS2 recruited from the Gargano area (Center East Coast of Italy)^27^,^28^,^29^ in whom both serum and RNA samples were available.

As per selection criteria, all subjects had fasting plasma glucose <126 mg/dL, were free from overt cardiovascular disease (i.e. by means of self-report) and were not treated with medications known to interfere with glucose homeostasis, insulin sensitivity and lipid levels. In all individuals clinical examination data were obtained from standardized interview, and blood sample collection were obtained between 8:00 and 9:00 AM after an overnight fast. Anthropometric and clinical features were obtained as previously described^29^. Briefly, in each subject (standing), waist circumference (the widest value between the lower rib margin and the iliac crest) was measured with a plastic measuring tape by the same investigator. Systolic and diastolic (disappearance of Korotkoff sound, phase V) blood pressure was measured in the sitting position with an appropriately sized cuff after a 5-min rest. Plasma glucose (mmol/l), serum insulin (pmol/l), and lipid profile (total serum cholesterol, HDL-cholesterol, serum triglycerides) were measured using commercially available enzymatic kits as previously described^30^. The insulin resistance index HOMA_IR_ was calculated as fasting serum insulin (pmol/liter) x fasting plasma glucose (mmol/liter)/135.

In GS2, IR/LGI-related CRF, including BMI, waist circumference, HOMA_IR_, HDL-cholesterol, triglycerides and systolic and diastolic blood pressure were considered as outcomes.

#### Gargano Heart Study (GHS)-prospective design

This sample included 359 patients with type 2 diabetes (American Diabetes Association 2003 criteria) and coronary artery disease, consecutively recruited at the Endocrine Unit of IRCCS “Casa Sollievo della Sofferenza” in San Giovanni Rotondo (Gargano, Center East Coast of Italy) from 2001 to 2008^16^,^17^,^31^,^32^, whose serum was available. All patients had either a stenosis > 50% in at least one coronary major vessel at coronary angiography or a previous myocardial infarction (MI). Follow-up information on outcomes was collected yearly from 2002 to 2011. The only exclusion criterion was the presence of poor life expectancy for non diabetes-related diseases. The end-point was a combination of MACE including cardiovascular deaths (i.e. according to the international classification of diseases’ codes: 428.1- ninth edition - and I21.0-I21.9, I25.9, I46.9-I50.9, I63.0, I63.9, I70.2- tenth edition), non-fatal MIs and non-fatal strokes. Confirmation of the event was obtained from death certificates. For all non-fatal MIs and strokes, confirmation of the events was obtained from the hospital medical records. Clinical data at baseline were obtained from a standardized interview and examination. Blood samples were obtained between 8:00 and 9:00 AM after an overnight fast and anthropometric and clinical features were obtained as previously described^16^,^17^,^31^,^32^. Smoking habits and history of hypertension (as indicated by the presence of anti-hypertension therapy), dyslipidemia (as indicated by the presence of anti-dyslipidemia therapy) and MI as well as glucose-lowering treatment were also recorded at time of examination. Data regarding medications were confirmed by review of medical records.

In the GHS-prospective design, the combination of above-described MACE were considered as the only outcome.

### Informed consent and ethics committee approval

Study design and informed consent procedures were approved by the local Institutional Ethic Committee of “Casa Sollievo della Sofferenza” Institute (N_4080/08, 4/8/2009) and performed according to the Helsinki Declaration. All participants gave written informed consent.

### RNA extraction and gene expression analyses

Total RNA from peripheral whole blood cells (PWBC) was extracted from whole blood specimens using PAXgene Blood RNA kit (PreAnalytiX, GmbH, Germany). RNA was eluted in RNAse free-water and stored at −80 °C until used. Total RNA yield and purity were determined spectrophotometrically using the NanoDrop ND-1000 (Wilmington, DE, USA). Integrity of resuspended total RNA was determined by electrophoretic separation and subsequent laser induced florescence detection using the RNA 6000 Nano Assay Chip Kit on the Bioanalyzer 2100 (Agilent Technologies, Waldbronn, Germany).

cDNA was generated by reverse transcription with iScript^TM^ Advanced cDNA Synthesis Kit (Biorad, Hercules, CA), according to the manufacturer’s instructions and used as template in the subsequent analyses^27^,^28^,^29^.

In order to make an accurate normalization of real time quantitative RT-PCR, we first measured the mRNA expression levels of 30 References Genes (Supplementary Table S1) on 150 cDNA samples randomly chosen. Expression of all 30 listed genes were quantitated by using sybr green assay on custom Reference Genes H384/HSR- plates (Biorad, Hercules, CA) and results were analyzed by using PrimePCR™ Analysis Software. Four genes, including *Pop4* (processing of precursor 4)*, Nono* (non-POU-domain-containing), *RPL13A* (non-POU-domain-containing) and *GUSB* (glucuronidase) with the best control gene-stability, measured as M value^33^, were finally chosen as housekeeping genes and used for subsequent analysis.

Expression levels of *RETN* (resistin), *IL1B*, *IL6*, *IL8, IL12A* (coding for the 35 kD cytokine receptor like subunit), *IL12B* (coding for the 40 kD cytokine receptor like subunit), and *TNFA* were measured by using specific sybr green assay on custom PrimePCR Assays and Controls plates (Biorad, Hercules, CA). The relative expression levels were calculated by using the comparative ΔCT method. Briefly, the amount of all genes run in duplicates was normalized to the geometric mean of the 4 chosen housekeeping genes, used as endogenous reference (2^−ΔCT^).

### Measurement of circulating cytokines

Serum resistin concentrations were measured in duplicate by a commercial ELISA (Bio Vendor, Brno Czech Republic) as previously described^34^. Inter- and intra-assay coefficients of variation were 7.0–8.1% and 5.2–6.6%, respectively. Serum IL-1β, IL-6, IL-8, IL-12 and TNF-α circulating levels were measured in duplicate, using multiplex detection 5-plex kit from Bio-Rad (Hercules, CA). Of note, IL-12 is a heterodimer composed of the 35 kD cytokine receptor like subunit encoded by IL-12A, and a 40 kD subunit encoded by IL-12B. The internal control consisted of serum from an admixture of blood samples from 12 GS2 participants. The median coefficient of variation (CV) was <25% for all analyzed cytokines. Data analyses were performed using Bio-Plex Manager software version 6.1 (Bio-Rad). Cytokine concentrations were interpolated from an appropriate standard curve.

### Statistical methods

Patients’ baseline characteristics were reported as mean ± SD and percentages for continuous and categorical variables, respectively. HOMA_IR_, triglycerides and all cytokines values were not normally distributed; therefore a natural log transformation was performed before further analyses. Expression and cytokines data were subtracted by their means and divided (standardized) by their SD, so that the β values, derived from linear regression models, represent the mean variation in the endpoint for 1 SD increment of the exposure of interest.

To assess the impact of collinearity among variables included into the multivariable model, variable inflation factors, which denote the factors by which the variance and the standard error of the estimated regression coefficients are inflated when multi-collinearity exists, were estimated as previously described^35^.

In order to consider both the expression and serum levels of the resistin-induced cytokines as a whole (i.e. resistin pathway), we created weighted expression RPSs (w-eRPSs), as well as weighted serum RPSs (w-sRPSs). Each w-eRPS was constructed as it follows: i) log-transformed and standardized expression data of all cytokines but *IL12B* were considered; ii) multivariable linear regression analyses for each outcome of interest, with all cytokines together as covariates, were performed to obtain the regression coefficients estimates (βs) for each cytokine. To reduce the risk of too optimistic discoveries, regression coefficients (weights) have been estimated following a bootstrap approach with 100,000 re-samplings with replacement; iii) each raw w-eRPS has been obtained as a sum of cytokine values, each weighted by its regression coefficient, i.e. (β1 × cytokine 1) + (β2 × cytokine 2) + (β3 × cytokine 3) and so on; iv) each final w-eRPS has been obtained after dividing the score by its SD. Each w-sRPS was calculated as described for w-eRPSs by using serum data naturally log-transformed and standardized of all cytokines but IL-12.

The w-sRPS in GHS-prospective design was created as previously described, by using as β values (weights) those obtained by fitting the multivariable proportional hazards Cox regression model for the combination of MACE. Also in this case, the risk of too optimistic discoveries was addressed by estimating regression coefficients following a bootstrap approach with 100,000 re-samplings with replacement. Then, the raw score was divided by its SD, as described before.

By taking into account the effect size of each cytokine, such scores allow to investigate the role of all cytokines as a whole (i.e. resistin pathway).

All β values as well as distribution of all w-RPSs are shown in Supplementary Tables S2 and S3 and Supplementary Figure S1.

Relationships among variables were evaluated by either univariable or multivariable linear regression analyses, as appropriate.

In the GHS-prospective design prediction analysis, the time variable was defined as the time between the baseline examination and date of the end-point of interest (i.e. MACE), or, for subjects who did not experience the event, the date of the last available clinical follow-up. Incidence rates for the endpoint of interest were expressed as the number of new events per 100 person years (py). Univariable and multivariable Cox proportional hazards regressions analyses were performed to assess the association between w-sRPS and the event occurrence. Risks were reported as HRs along with their 95% CI for SD increase in w-sRPS. A p-value < 0.05 was considered as statistically significant. All analyses were performed using SPSS v.15 (SPSS, Chicago IL) and SAS Release 9.3 (SAS Institute, Cary, NC).

## Results

Clinical features of 280 healthy individuals from GS2, as well as those of 359 patients with type 2 diabetes from the GHS-prospective design, are reported in Table 1.

### Exploring the plausibility of a resistin-pathway

In order to explore the plausibility of a multi-cytokine resistin pathway in human beings, the relationships between resistin and IL-1β, IL-6, IL-8, IL-12 and TNF-α levels (both in terms of mRNA expression in PWBC and serum concentration) were explored in healthy individuals from GS2.

All 280 study-subjects from GS2 had detectable cytokine expression levels. Using linear regression analyses, resistin levels were significantly correlated with those of all cytokines, but *IL12B* (coding for the 40 kD cytokine receptor like subunit) (Table 2).

When assessing serum cytokine concentrations in the same healthy individuals, 57 (20.4%), 12 (4.3%), 53 (19%) and 70 (25%) had IL-1, IL-6, IL-12 and TNF-α levels below detection limits and could not, therefore, used for further analyses (Supplementary Figure S2). Thus, the available study sample for serum data comprised 187 individuals. Clinical features of these 187 individuals were totally superimposable to those of the whole initial sample (Supplementary Table S4). Similarly and coherently to what observed with expression levels, serum resistin concentrations were strongly and significantly associated with serum concentrations of all cytokines, but IL-12 (Table 2). Of note, no evidence of multi-collinearity was observed, as indicated by a variable inflation factor value of less than 5^35^. This speaks against the possibility that our results have been affected by the observed correlation between resistin and most cytokines.

Taken together, these data speak in favor for the existence in humans of a resistin pathway, which, at least partly, is regulated at transcriptional level.

Based on such findings, we created two multi-marker resistin pathway scores, by simply summing either expression (eRPS) or serum levels (sRPS) of all cytokines but *IL12B* and IL-12, respectively. Of note, eRPS was directly and significantly related to sRPS (r = 0.27, p = 2.2 × 10^–4^, by Spearman correlation), thus further suggesting a transcriptional regulation of the serum resistin pathway, considered as a whole.

### Resistin pathway and IR/LGI-related cardiovascular risk factors

In order to investigate whether or not the above-proposed resistin pathway was associated with cardiovascular risk factors related to IR and LGI, weighted sRPS (w-sRPSs), as well as a weighted eRPS (w-eRPSs) data were created for each outcome of interest, as described in Methods. The distribution of such scores in the GS2 is shown in Supplementary Figure S1, panel A.

As shown in Table 3, w-eRPSs were significantly associated with all cardiovascular risk factors related to IR and LGI, but systolic blood pressure. Similar results were obtained when sex, age and BMI were considered as covariates into the models (Table 3).

Similarly, w-sRPSs were strongly and significantly associated with cardiovascular risk factors related to IR and LGI, in both the unadjusted and the adjusted models (Table 4).

In both cases, the association with waist circumference was no longer significant after adjusting for BMI.

In contrast to what observed with both w-eRPSs and w-sRPSs, in a multivariable analysis comprising all individual cytokines, only scattering associations with IR/LGI-related cardiovascular risk factors were observed (Supplementary Tables S5 and S6), thus suggesting that the two scores drive additional, more comprehensive, information, as compared to that of each single cytokine.

### sRPS and MACE

In the GHS-prospective design, during follow-up (5.4 ± 2.5 years), 71 MACE (i.e. 58 cardiovascular deaths, 6 non-fatal MIs and 7 non-fatal strokes) occurred, corresponding to an overall annual incidence rate of 3.7% (71 events/1,906 py).

As expected, serum concentration of all cytokines was much higher in this sample, comprising patients with type 2 diabetes, as compared to healthy controls from the GS2 (p < 0.01 for all). In fact, among GHS-prospective design participants, only 2 (0.6%), 4 (1.1%), 3 (0.8%) and 3 (0.8%) had IL-1, IL-6, IL-8 and TNF-α serum levels below detection limits so that, in this sample 353 out of 359 participants (Supplementary Figure S2) were available for further analyses. In this cohort, a significant association was observed between serum resistin and cytokines belonging to RPS, as indicated by data obtained in GS2 (r^2^ ranging 0.04–0.08; p values ranging 6.5 × 10^−8^−9.3 × 10^−5^), but TNF-α. Despite this, because of our pre-specified study design (i.e. aimed at testing the role of RPS, as obtained in healthy individuals, on MACE), we created a weighted multi-marker serum resistin pathway score (w-sRPS) using the same five cytokines as in GS2, thus preferring a conservative, rather than an opportunistic, data-driven (i.e. without TNF-α values) approach. Distribution of such score is shown in Supplementary Figure S1, panel B.

For each SD increase of w-sRPS the HR (95% CI) for MACE was 1.60 (1.30–1.96), Table 5. The observed association did not change much after adjusting for sex, age and smoking habits (HR, 95% CI = 1.71, 1.37–2.13). Similar results were observed when also BMI, HbA1_C_, insulin treatment as well as anti-hypertension and anti-dyslipidemia therapies and hsCRP were considered (HR, 95% CI = 1.44, 1.10–1.90, Table 5), with HbA1_C_ being among those added into the model, the only other risk factor remaining significantly associated with MACE (Table 5). Given the established relationship of serum resistin with MACE^10^,^11^,^14^,^15^,^16^,^17^,^18^,^20^,^21^, that we here in fact further confirm (HR, 95% CI = 1.37, 1.07–1.74) in a multivariable analysis comprising all cytokines belonging to the resistin pathway (Supplementary Table S7), the association between w-sRPS and MACE was adjusted by resistin levels; in such a model, the mean effect of w-sRPS did not change at all (HR for each SD increase = 1.60, 95% CI = 1.07–2.39), while that of resistin itself was no longer significant (HR, 95% CI = 0.99, 0.65–1.52), thus indicating that in the context of MACE, w-sRPS drives additional, more comprehensive, information than resistin alone.

## Discussion

Previous experimental evidences indicate that, in cellular and animal models, the expression of several cytokines, including IL-1β, IL-6, IL-8 IL-12 and TNF-α, is under the control of resistin^22^,^23^,^24^,^25^,^26^. These evidences point to the existence of a multi-cytokine resistin pathway and make possible to hypothesize that it exerts, as a whole, a deleterious role on IR and LGI.

Whether a resistin pathway does exist in humans and, if so, whether it does play a detrimental role on IR/LGI-related clinical outcomes has never been addressed, with only sparse data on the correlation between resistin and some of the above-mentioned cytokines^36^,^37^,^38^,^39^,^40^,^41^ being so far available. Thus, by means of epidemiological data, we tested the hypothesis that in human beings a resistin pathway does exist and does have a role on cardiovascular disease.

To the best of our knowledge, our study is the first clearly pointing to such a pathway in human beings, which, according to our correlative data, comprises IL-1β, IL-6, IL-8 and TNF-α but not IL-12. This latter cytokine, (also known as IL-12p70) is the protein product of *IL12A* (coding for the IL-12 35 kD subunit) and *IL12B* (coding for the IL-12 40 kD subunit). Thus, in the absence of correlation between mRNA levels of resistin and *IL12B*, it is conceivable that, in the same individuals, no correlation was observed also between serum resistin and IL-12 concentrations. Our data seem to be in contrast with those by Silswal *et al*. reporting the ability of recombinant resistin to stimulate IL-12 secretion in human and mouse cultured macrophages^25^. Differences in the study design, including only *in vitro* conditions and the lack of expression data in Silswal’s study^25^, may well explain the apparent discrepancy.

In general, obtaining consistent results across two different biological specimens increases their credibility. In the specific context of our data, such consistency clearly suggests that serum cytokine levels are, at least partly, under transcriptional control, with PWBC resulting an easy-obtainable and reliable tool, as reported in previous cytokine studies^16^,^42^. Also the correlation between eRPS and sRPS, though the latter is likely to be the net result of several additional metabolic and clearance processes, speaks in favor of a transcriptional control of serum cytokines levels.

Entirely novel is also the finding that both w-eRPS and w-sRPS are significantly and independently associated with several cardiovascular risk factors related to IR and LGI. It is of note, that, as previously reported in the general population^43^,^44^,^45^, when singly considered, only some cytokines, were variably associated with only some cardiovascular risk factors, thus suggesting that the two scores we utilized do sign comprehensively a biological pathway providing information which is not entirely derivable from any single cytokine. These associations were obtained in healthy individuals taking no medications, thus making sure that our results are not biased by either concomitant overt diseases and/or organ damages, or by ongoing treatments. Due to the lack of information on smoking habit, we cannot exclude it has a confounder role on the associations of w-eRPS and w-sRPS with cardiovascular risk factors.

An additional novel and utmost important finding of our study is that w-sRPS strongly predicts incident MACE in type 2 diabetes, independently of several covariates, including hsCRP, the most established marker of LGI and smoking habit, thus making unlikely this latter risk factor has played a role on the association with cardiovascular risk factors. In addition, this association does not change when also resistin is considered as a covariate, thus further evidencing that the whole pathway drives a comprehensive effect of all study cytokines which adds on top of that driven by any cytokine alone. Such a scenario is also suggested by the difference in the HR values for MACE, being equal to 1.37 and to 1.60 for resistin and sRPS, respectively, which, though not reaching statistical significance, represents a 67% mean effect size increase on the log scale which, if confirmed in further studies, is not a trivial improvement.

Of note, in the multivariable model we tested, both w-sRPS and HbA1_C_ were significantly associated with MACE, thus suggesting that they sign different pathways in affecting cardiovascular risk.

### Strengths and limitations

The major strength of our study is its novelty. In fact, although the correlation between resistin and each single cytokine we investigated had been previously reported, this is the first study supporting the existence of a resistin pathway in humans, which has a role on IR/LGI-related cardiovascular risk factors and MACE.

A further strength is that the associations between serum resistin pathway and cardiovascular risk factors observed in healthy individuals from GS2, is fully coherent with the association with extremely relevant clinical outcomes, namely MACE, in high-risk individuals from the GHS-prospective design. Such high consequentiality across two totally different study samples increases the overall credibility of our findings.

Finally, it is also of note that we provide some insights about the control of serum cytokine concentrations which our data suggest to be partly determined at transcriptional level.

A major limitation of our finding is due to the intrinsic nature of correlative studies like ours, which, by definition, cannot address the biology underlying the reported associations. However, the well-known deleterious effects of all components of the resistin pathway on both IR and LGI^4^,^5^ do represent a credible and strong biological background for the data we here report. Also, the correlative nature of our data does not allow us to test whether some of the cytokines comprised in the resistin pathway control, in turn, resistin expression and/or concentration, in the context of an interwoven relationship.

As a second limitation, we recognize that our expression data were not obtained in the most relevant cell types for cytokine production and low-grade inflammation processes, namely adipocytes and/or adipose tissue infiltrating macrophages. However, one should note that PWBC i) is an easily accessible and established model for studying gene expression profiling^46^,^47^,^48^, ii) comprise also macrophages’ precursor mononuclear cells which do have a relevant role on the above-mentioned pathogenic processes, and iii) have been successfully utilized in the context of cytokine gene expression changes in cardiovascular risk related to IR and LGI^16^,^27^,^47^,^48^. Moreover, despite circulating cytokine concentrations are widely used in epidemiological studies, it should be kept in mind that they are not necessarily representative of the paracrine mechanisms, ongoing in specific cells and tissues relevant for cardiovascular disease.

In addition, we acknowledge that the association with MACE was investigated in a prototype of high-risk individuals, as patients with type 2 diabetes and established coronary artery disease have to be certainly considered. Understanding whether a similar association is observable also in other subtypes of high-risk individuals (including obese in whom adipose tissue dysfunction is linked to increased cardiovascular risk) and/or in the general population deserves further, specifically designed studies. Also unknown is if our finding is extendable to individuals with different environmental and/or genetic backgrounds. Finally, still focusing on the extendibility of our results, we acknowledge that, due to the lack of data, the possible role of different estrogen levels in women of different age have not been addressed.

Lack of validation of our score in an independent population is a further limitation of our study. In a broader sense, a replication of our finding in different clinical sets and in different ethnicities would reinforce its clinical meaning.

## Conclusions

**Figure 1:**
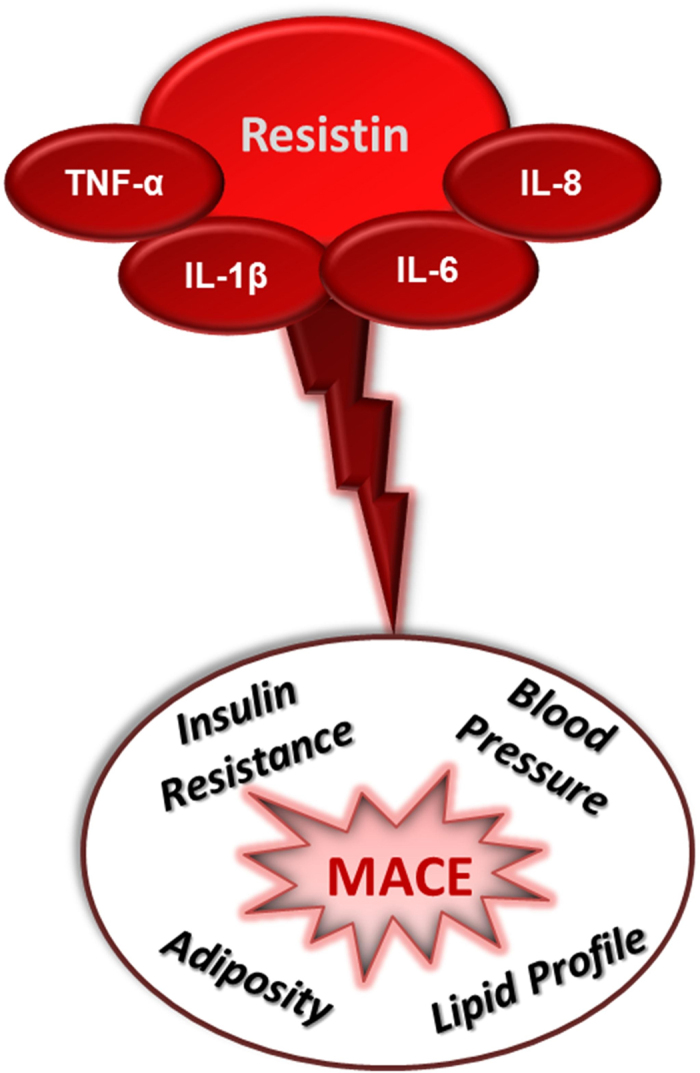
Role of a multi-cytokine resistin pathway on cardiovascular disease. Our correlative data, together with previous findings reporting in cultured cells a direct effect of resistin on the expression of IL-1β, IL-6, IL-8 and TNF-α, are compatible with the existence of a multi-cytokine resistin pathway which is associated with several IR/LGI-related risk factors and major adverse cardiovascular events (MACE).

To the best of our knowledge, this is the first study reporting data fully compatible with the existence of a multi-cytokine resistin pathway, which predicts cardiovascular disease (Fig. 1). Such a role is independent of several covariates, including hsCRP, currently the most established inflammatory marker utilized in the clinical set.

If our finding is confirmed in additional larger studies, it would be important to verify whether adding the whole resistin pathway to validated risk engines improves MACE prediction, thus becoming of clinical relevance.

## Additional Information

**How to cite this article:** Menzaghi, C. *et al*. Suggestive evidence of a multi-cytokine resistin pathway in humans and its role on cardiovascular events in high-risk individuals. *Sci. Rep.* **7**, 44337; doi: 10.1038/srep44337 (2017).

**Publisher's note:** Springer Nature remains neutral with regard to jurisdictional claims in published maps and institutional affiliations.

## Supplementary Material

Supplementary Information

## Figures and Tables

**Table 1:** Clinical characteristics of participants from GS2 and GHS-prospective design.

	GS2 (n = 280)	GHS (n = 359)
Sex (M/F)	224/56	242/117
Age (yrs)	42.8 ± 11.4	64.4 ± 8.1
BMI (Kg/m^2^)	27.1 ± 3.5	30.2 ± 4.8
Waist circumference (cm)	93.9 ± 10.6	NA
HOMA_IR_	2.0 ± 1.6	NA
Triglycerides (mg/dl)	130.4 ± 94.2	152.6 ± 91.8
HDL-Cholesterol (mg/dl)	50.4 ± 11.0	43.6 ± 14.6
SBP (mm Hg)	125.6 ± 10.8	134.9 ± 19.1
DPB (mm Hg)	79.8 ± 6.7	76.4 ± 9.8
Smokers (%)	NA	64 (17.8)
Diabetes duration (yrs)	NA	13.8 ± 9.2
HbA_1C_ (% and mmol/mol)	NA	8.6 ± 1.9 and 70.8 ± 20.6
Insulin w or w/o oral agents (%)	NA	194 (54.0)
Anti-hypertension therapy (%)	NA	305 (85.0)
Anti-dyslipidemia therapy (%)	NA	233 (64.9)
hsCRP (mg/L)	NA	1.4 (0.7–5.5)

Continuous variables were reported as mean ± SD, whereas categorical variables as total frequency.

GS2: Gargano Study 2; M: males; F: females; BMI: body mass index; HOMA_IR_: Homeostatic model assessment of insulin resistance; SBP: systolic blood pressure; DBP: diastolic blood pressure. HbA_1c_: glycated haemoglobin A1_C_; hsCRP: high sensitive C reactive protein. NA: not applicable.

**Table 2:** Correlation between Resistin and other cytokines, possibly belonging to “resistin pathway”.

	Gene expression			Serum concentration		
	β	r^2^	p	β	r^2^	p
**IL-1B**	0.45	0.19	3.5 × 10^−14^	0.34	0.12	1.5 × 10^−6^
**IL-6**	0.23	0.05	1.1 × 10^−4^	0.38	0.15	3.1 × 10^−8^
**IL-8**	0.39	0.15	1.6 × 10^−11^	0.51	0.26	6.0 × 10^−14^
**IL-12**				−0.03	0.001	0.73
IL-12A	0.21	0.045	3.3 × 10^−4^			
IL-12B	−0.01	0.001	0.88			
**TNFA**	0.37	0.14	1.1 × 10^−10^	0.18	0.03	2.4 × 10^−2^

β values, derived from linear regression analyses, represent the change of either cytokines expression or serum levels for 1 SD resistin increase.

IL12 is a heterodimer composed of the 35 kD cytokine receptor like subunit encoded by IL-12A, and a 40 kD subunit encoded by IL-12B, thus, for gene expression, measurements of both genes were assessed.

**Table 3:** Correlation between w-eRPSs and IR/LGI-related cardiovascular risk factors in GS2.

	β	r^2^	p	p*	p§
BMI	0.79	0.05	1.3 × 10^−4^	4.4 × 10^−4^	
WAIST	2.27	0.05	3.0 × 10^−4^	5.3 × 10^−3^	0.23
Ln HOMA_IR_	0.11	0.04	1.3 × 10^−3^	1.6 × 10^−3^	2.3 × 10^−3^
Ln TG	0.09	0.03	2.5 × 10^−3^	1.6 × 10^−3^	1.2 × 10^−3^
HDL-Cholesterol	4.41	0.03	2.4 × 10^−3^	2.1 × 10^−2^	3.3 × 10^−2^
SBP	1.16	0.01	0.08	0.11	0.11
DBP	1.59	0.06	6.9 × 10^−5^	2.3 × 10^−4^	7.3 × 10^−4^

^*^Sex and age adjusted.

^§^BMI, sex and age adjusted.

w-eRPSs: weighted expression resistin pathway scores; IR/LGI: insulin resistance/low grade inflammation; GS2: Gargano Study 2; BMI: body mass index; HOMA_IR_: Homeostatic model assessment of insulin resistance; SBP: systolic blood pressure; DBP: diastolic blood pressure. β values derived from linear regression analyses represent the change of each risk factors, expressed in unit, for 1 SD increase in w-eRPSs.

**Table 4:** Correlation between w-sRPSs and IR/LGI-related cardiovascular risk factors in GS2.

	β	r^2^	p	p*	p§
BMI	0.82	0.05	2.0 × 10^−3^	5.6 × 10^−3^	
WAIST	2.34	0.05	3.0 × 10^−3^	1.5 × 10^−2^	0.52
Ln HOMA_IR_	0.13	0.06	5.8 × 10^−4^	5.1 × 10^−4^	2.1 × 10^−4^
Ln TG	0.13	0.07	3.9 × 10^−4^	3.3 × 10^−4^	3.9 × 10^−4^
HDL-Cholesterol	4.43	0.16	2.0 × 10^−8^	1.8 × 10^−8^	1.5 × 10^−7^
SBP	2.85	0.06	5.8 × 10^−4^	4.6 × 10^−4^	4.7 × 10^−4^
DBP	1.64	0.07	2.6 × 10^−4^	6.1 × 10^−4^	1.0 × 10^−3^

^*^Sex and age adjusted.

^§^BMI, sex and age adjusted.

w-sRPSs: weighted serum resistin pathway scores; IR/LGI: insulin resistance/low grade inflammation; GS2: Gargano Study 2; BMI: body mass index; HOMA_IR_: Homeostatic model assessment of insulin resistance; SBP: systolic blood pressure; DBP: diastolic blood pressure. β values derived from linear regression analyses represent the change of each risk factors, expressed in unit, for 1 SD increase in w-sRPSs.

**Table 5:** Univariable and multivariable associations between w-sRPS, cardiovascular risk factors and MACE in GHS-prospective design.

	Univariable		Multivariable	
	HR (95% CI)	p	HR (95% CI)	p
w-sRPS (per 1 SD increase)	1.60 (1.30–1.96)	9.0 × 10^−6^	1.44 (1.10–1.90)	9.0 × 10^−3^
Males vs Females	1.45 (0.85–2.45)	0.17	1.81 (0.93–3.56)	0.08
Age at recruitment (per 1 yr)	1.05 (1.01–1.07)	5.0 × 10^–3^	1.03 (0.99–1.07)	0.11
Smoking habits (yes *vs.* no)	0.97 (0.49–1.92)	0.93	1.00 (0.54–2.43)	0.99
BMI (per 1 unit)	0.94 (0.88–1.00)	4 × 10^−2^	0.96 (0.90–1.02)	0.15
HbA1c (per 1 unit)	1.16 (1.03–1.30)	1.1 × 10^−2^	1.22 (1.07–1.41)	4.0 × 10^−3^
Insulin therapy (yes *vs.* no)	2.34 (1.37–4.00)	2.0 × 10^−2^	1.83 (1.01–3.32)	5.0 × 10^−2^
Anti-hypertension therapy (yes *vs.* no)	1.09 (0.54–2.20)	0.80	1.70 (0.72–4.00)	0.22
Anti-dyslipidemia therapy (yes *vs.* no)	0.62 (0.38–1.00)	0.06	0.88 (0.48–1.62)	0.69
hsCRP (per 1 SD increase)	1.60 (1.28–2.00)	2.9 × 10^−5^	1.31 (0.96–1.80)	0.09

w-sRPSs: weighted serum resistin pathway scores; MACE: major adverse cardiovascular events; GHS: Gargano Heart Study. BMI: body mass index; hsCRP (high sensitive C reactive protein). HbA_1c_: glycated haemoglobin A1_c_.
