# Supplementary material for: Suggestive evidence of a multi-cytokine resistin pathway in humans and its role on cardiovascular events in high-risk individuals
Source: Sci Rep. 2017 Mar 14;7:44337. doi: 10.1038/srep44337 (PMC5349527; doi:10.1038/srep44337)
Supplement: Supplementary Information [file srep44337-s1.pdf]

Suggestive evidence of a multi-cytokine resistin pathway in humans and its role on cardiovascular events in high-risk individuals

Claudia Menzaghi[1], Antonella Marucci[1], Alessandra Antonucci[1], Concetta De Bonis[1], Lorena Ortega Moreno[1^], Lucia Salvemini[1], Massimiliano Copetti[2], Vincenzo Trischitta[1,3*], Rosa Di Paola[1*]

SUPPLEMENTARY INFORMATION

Supplementary Table S1. List of reference genes evaluated in 150 subjects randomly chosen from GS2.

Gene Name/ Gene Symbol	Unique Assay ID
actin, beta / ACTB	qHsaCED0036269
aminolevulinate, delta-, synthase 1 / ALAS1	qHsaCID0009300
beta-2-microglobulin / B2M	qHsaCID0015347
cyclin-dependent kinase inhibitor 1A (p21, Cip1) / CDKN1A	qHsaCID0014498
glucose-6-phosphate dehydrogenase / G6PD	qHsaCED0001353
glyceraldehyde-3-phosphate dehydrogenase / GAPDH	qHsaCED0038674
glucuronidase, beta / GUSB	qHsaCID0011706
hemoglobin, beta / HBB	qHsaCED0020657
hydroxymethylbilane synthase/ HMBS	qHsaCID0038839
hypoxanthine phosphoribosyltransferase 1 / HPRT1	qHsaCID0016375
heat shock protein 90kDa alpha (cytosolic), class B member 1 / HSP90AB1	qHsaCED0038850
importin 8 / IPO8	qHsaCED0005354
lactate dehydrogenase A / LDHA	qHsaCED0001212
non-POU domain containing, octamer-binding / NONO	qHsaCED0002050
phosphoglycerate kinase 1 / PGK1	qHsaCED0003721
processing of precursor 4, ribonuclease P/MRP subunit (S. cerevisiae) / POP4	qHsaCID0015127
peptidylprolyl isomerase A (cyclophilin A) / PPIA	qHsaCED0038620
peptidylprolyl isomerase H (cyclophilin H) / PPIH	qHsaCID0038169
proteasome (prosome, macropain) 26S subunit, ATPase, 4 / PSMC4	qHsaCID0011519
pumilio homolog 1 (Drosophila) / PUM1	qHsaCID0010952
ribosomal protein L13a / RPL13A	qHsaCED0020417
ribosomal protein L30 / RPL30	qHsaCED0038096
ribosomal protein, large, P0 / RPLP0	qHsaCED0038653
ribosomal protein S17 / RPS17	qHsaCED0041771
ribosomal protein S18 / RPS18	qHsaCED0037454
succinate dehydrogenase complex, subunit A, flavoprotein (Fp) / SDHA	qHsaCED0038853
TATA box binding protein / TBP	qHsaCID0007122
transferrin receptor (p90, CD71) /TFRC	qHsaCID0022106
ubiquitin C / UBC	qHsaCED0023867
tyrosine 3-monooxygenase/tryptophan 5-monooxygenase activation protein, zeta polypeptide / YWHAZ	qHsaCID0013897

Unique Assay ID. This is a unique identifier that can be used to identify the assay in the literature and online at www.bio-rad.com/.

Supplementary Table S2. Multivariable regression coefficients estimates of cytokine expression levels for IR/LGI-related cardiovascular risk factors in GS2.

	RETN		***IL1***		***IL6***		***IL8***		***IL12A***		***TNFA***	
	**β**	**95% (CI)**	**β**	**95% (CI)**	**β**	**95% (CI)**	**β**	**95% (CI)**	**β**	**95% (CI)**	**β**	**95% (CI)**
**BMI**	-0.26	(-0.72-0.18)	0.75	(0.32-1.21)	-0.05	(-0.94-0.85)	-0.31	(-1.13-0.42)	0.34	(-0.49-1.25)	0.45	(0.01-0.88)
**WAIST**	-0.05	(-1.40-1.22)	1.20	(-0.06-2.54)	-1.23	(-4.00-1.37)	0.03	(-2.37-2.22)	0.36	(-2.25-3.37)	1.75	(0.45-3.03)
**LnHOMA**	-0.05	(-0.12-0.02)	0.06	(-0.02-0.14)	-0.22	(-0.42- -0.04)	0.02	(-0.12-0.16)	0.19	(-0.01 0.42)	-0.01	(-0.08-0.06)
**Ln TG**	0.07	(-0.01-0.15)	-0.04	(-0.12-0.03)	0.01	(-0.14-0.14)	0.03	(-0.09-0.15)	0.02	(-0.12-0.18)	-0.03	(-0.10-0.04)
**HDL-Cholesterol**	-1.88	(-3.55- -0.26)	-0.17	(-1.85-1.50)	-0.36	(-3.31-2.51)	0.12	(-2.52-2.79)	-0.32	(-3.43-2.55)	0.38	(-1.03-1.86)
**SBP**	0.55	(-1.07-2.12)	0.27	(-1.07-1.63)	-1.92	(-5.33-1.16)	0.61	(-2.24-3.90)	0.89	(-2.42-4.55)	-0.42	(-1.87-1.06)
**DBP**	0.81	(-0.04-1.65)	0.68	(-0.17-1.56)	0.32	(-1.54-2.15)	-1.10	(-2.54-0.38)	0.01	(-1.73-1.75)	0.90	(0.14-1.71)

IR/LGI: insulin resistance/low grade inflammation; GS2: Gargano Study 2; BMI: body mass index; $HOMA_{IR}$: Homeostatic model assessment of insulin resistance; SBP: systolic blood pressure; DBP: diastolic blood pressure.

β: multivariable linear regression coefficients of each cytokine, expressed in unit, for the outcomes of interest. To reduce the risk of too optimistic discoveries, regression coefficients have been estimated following a bootstrap approach with 100,000 re-samplings, with replacement as described in Methods.

Supplementary Table S3. Multivariable regression coefficients estimates of cytokine concentration for IR/LGI-related cardiovascular risk factors in GS2 and for MACE in GHS-prospective design.

	**Resistin**		**IL-1β**		**IL-6**		**IL-8**		**TNF-α**	
	**β**	**95% (CI)**	**β**	**95% (CI)**	**β**	**95% (CI)**	**β**	**95% (CI)**	**β**	**95% (CI)**
**BMI**	0.30	-0.24±0.86	0.12	-0.86±1.04	1.40	0.36±2.58	-1.06	-1.90±-0.27	-0.51	-1.25±0.12
**WAIST**	1.28	-0.34±2.98	0.08	-2.77±2.75	3.59	0.15±7.31	-3.03	-5.87±-0.13	-0.52	-2.95±1.70
**LnHOMA**	-0.01	-0.09±0.06	-0.03	-0.15±0.10	-0.07	0.25±0.09	-0.05	-0.17±0.08	0.13	0.03±0.24
**Ln TG**	0.11	0.03±0.20	-0.03	-0.14±0.08	-0.01	-0.14±0.10	0.05	-0.06±0.16	0.02	-0.07±0.12
**HDL-Cholesterol**	-3.38	-5.01±-1.70	1.42	-0.91±3.81	-5.35	-8.73±-2.14	2.18	-0.15±4.56	1.67	-0.52±3.87
**SBP**	2.71	0.79±4.65	-2.08	-4.83±0.70	0.51	-3.19±3.95	-1.75	-4.56±1.08	1.21	-1.28±3.60
**DBP**	0.01	-1.08±1.09	-1.05	-2.71±0.61	1.82	-0.40±4.06	0.53	-1.53±2.58	0.30	-0.99±1.68
**MACE**	0.32	(0.07-0.60)	0.11	(-0.29-0.52)	0.23	(-0.17-0.61)	-0.002	(-0.34-0.34)	-012	(-0.45-0.26)

IR/LGI: insulin resistance/low grade inflammation; GS2: Gargano Study 2; MACE: major adverse cardiovascular events; GHS: Gargano Heart Study; BMI: body mass index; $HOMA_{IR}$: Homeostatic model assessment of insulin resistance; SBP: systolic blood pressure; DBP: diastolic blood pressure;

β: multivariable linear regression coefficients of each cytokine, expressed in unit, for the outcomes of interest. To reduce the risk of too optimistic discoveries, regression coefficients have been estimated following a bootstrap approach with 100,000 re-samplings, with replacement as described in Methods.

Supplementary Table S4. Baseline clinical features of participants from the GS2 in whom all serum cytokines levels were available.

**Sex (M/F)**	149/38
**Age (yrs)**	43.0±11.4
**BMI (Kg/m^2)**	27.1±3.6
**Waist circumference (cm)**	94.1±11.0
**HOMA$_{IR}$**	1.9±1.1
**Triglycerides (mg/dl)**	125.3±93.2
**HDL-Cholesterol (mg/dl)**	50.4±11.2
**SBP (mmHg)**	125.9±11.3
**DPB (mmHg)**	80.3±6.1

GS2: Gargano Study 2. Continuous variables were reported as mean ± SD, whereas categorical variables are total frequency. M: males; F: females; BMI: body mass index; HOMA$_{IR}$: Homeostatic model assessment of insulin resistance; SBP: systolic blood pressure; DBP: diastolic blood pressure.

Supplementary Table S5. Multivariable analyses between expression cytokine levels and IR/LGI-related cardiovascular risk factors in GS2.

	RETN		***IL1***		***IL6***		***IL8***		***IL12A***		***TNFA***	
	**β**	**p**	**β**	**p**	**β**	**p**	**β**	**p**	**β**	**p**	**β**	**p**
**BMI**	-0.26	0.30	0.75	3.0×10^{-2}	-0.05	0.92	-0.32	0.40	0.35	0.50	0.45	5.0×10^{-2}
**WAIST**	-0.04	0.96	1.18	0.13	-1.22	0.43	0.03	0.98	0.36	0.82	1.76	1.0×10^{-2}
**Ln HOMA**	-0.05	0.20	0.06	0.15	-0.22	1.0×10^{-2}	0.02	0.75	0.19	3.0×10^{-2}	-0.01	0.89
**Ln TG**	0.08	4.0×10^{-2}	-0.05	0.22	0.01	0.94	0.03	0.61	0.02	0.79	-0.03	0.40
**HDL-Cholesterol**	-1.89	2.0×10^{-2}	-0.14	0.86	-0.35	0.83	0.13	0.91	-0.36	0.83	0.39	0.59
**SBP**	0.58	0.47	0.24	0.76	-1.96	0.23	0.67	0.58	0.97	0.56	-0.42	0.57
**DBP**	0.83	0.09	0.67	0.17	0.29	0.77	-1.08	0.14	0.05	0.96	0.92	4.0×10^{-2}

IR/LGI: insulin resistance/low grade inflammation; GS2: Gargano Study 2; BMI: body mass index; $HOMA_{IR}$: Homeostatic model assessment of insulin resistance; SBP: systolic blood pressure; DBP: diastolic blood pressure.

The association with each single risk factor of all cytokines was tested by multivariable linear regression analyses: each row corresponds to a multivariable model whose specific outcome of interest is reported in the first column; β values represent the change in IR/LGI-related risk factors for 1 SD increase in expression cytokine levels.

Supplementary Table S6. Multivariable analyses between serum cytokine concentrations and IR/LGI-related cardiovascular risk factors in GS2.

	Resistin		IL-1		IL-6		IL-8		TNF-α	
	**β**	**p**	**β**	**p**	**β**	**p**	**β**	**p**	**β**	**p**
**BMI**	0.30	0.33	0.11	0.82	1.41	$1.0x10^{-2}$	-1.05	$3.0x10^{-2}$	-0.51	0.19
**WAIST**	1.28	0.18	0.06	0.97	3.57	$3.0x10^{-2}$	-2.97	$5.0x10^{-2}$	-0.50	0.68
**LnHOMA**	-0.02	0.73	-0.03	0.64	-0.07	0.41	-0.05	0.45	0.13	$2.0x10^{-2}$
**Ln TG**	0.11	$7.0x10^{-3}$	-0.04	0.58	-0.01	0.86	0.05	0.45	0.02	0.74
**HDL-Cholesterol**	-3.36	$1.0x10^{-4}$	1.44	0.32	-5.37	$1.0x10^{-3}$	2.11	0.14	1.69	0.15
**SBP**	2.71	$7.0x10^{-4}$	-2.15	0.17	0.53	0.76	-1.72	0.27	1.25	0.32
**DBP**	0.02	0.97	-1.04	0.21	1.81	$5.0x10^{-2}$	0.54	0.52	0.32	0.64

IR/LGI: insulin resistance/low grade inflammation; GS2: Gargano Study 2; BMI: body mass index $HOMA_{IR}$: Homeostatic model assessment of insulin resistance; SBP: systolic blood pressure; DBP: diastolic blood pressure.

The association with each single risk factor of all cytokines was tested by multivariable linear regression analyses; each row corresponds to a multivariable model whose specific outcome of interest is reported in the first column; β values represent the change in each in IR/LGI-related risk factors for 1 SD increase of log transformed serum cytokine concentration.

Supplementary Table S7. Multivariable association between serum cytokine concentrations and MACE in GHS-prospective design.

	**HR (95% CI)**	**p**
**Resistin**	1.37 (1.07-1.74)	$1.0x10^{-2}$
**IL-1**	1.13 (0.81-1.56)	0.49
**IL-6**	1.26 (0.89-1.78)	0.20
**IL-8**	0.99 (0.72-1.36)	0.95
**TNF-α**	0.88 (0.63-1.22)	0.43

MACE: major adverse cardiovascular events; GHS: Gargano Heart Study. HR (95% CI) are given for the increase of 1 SD of log transformed serum cytokine concentrations.

SUPPLEMENTARY FIGURES

Supplementary Figure S1. Distribution of w-eRPSs (Panel A) and w-sRPSs (Panel B).

Both w-sRPSs and w-eSRPs were calculated as described in Methods. Briefly: i) expression and serum data were rescaled; ii) bootstrapped multivariable linear regression analyses for each outcome were performed to obtained the regression coefficients estimates (βs) for each cytokine; iii) each raw w-RPS has been obtained as (β1×cytokine 1)+(β2×cytokine 2)+(β3×cytokine 3) and so on; iv) each final w-RPS has been obtained after rescaling the score by its SD.

Panel A shows the distribution of w-eRPSs for each IR/LGI-related cardiovascular risk factor in GS2.

Panel B shows the distribution of w-sRPSs for each IR/LGI-related cardiovascular risk factor in GS2 and MACE in GHS- prospective design.

BMI: body mass index; $HOMA_{IR}$: Homeostatic model assessment of insulin resistance; SBP: systolic blood pressure; DBP: diastolic blood pressure

Supplementary Figure S2. Box plots of cytokine levels in participants from GS2 (light grey) and GHS-prospective design (dark gray). The bottom and top of each box represents lower and upper quartile, respectively; the bottom and top of each dashed line indicates the minimum and maximum value, respectively; the line inside each box represents the median value. For each study sample, number and (percentage) of subjects, in whom cytokine levels were detectable, are indicated.

Figure S1

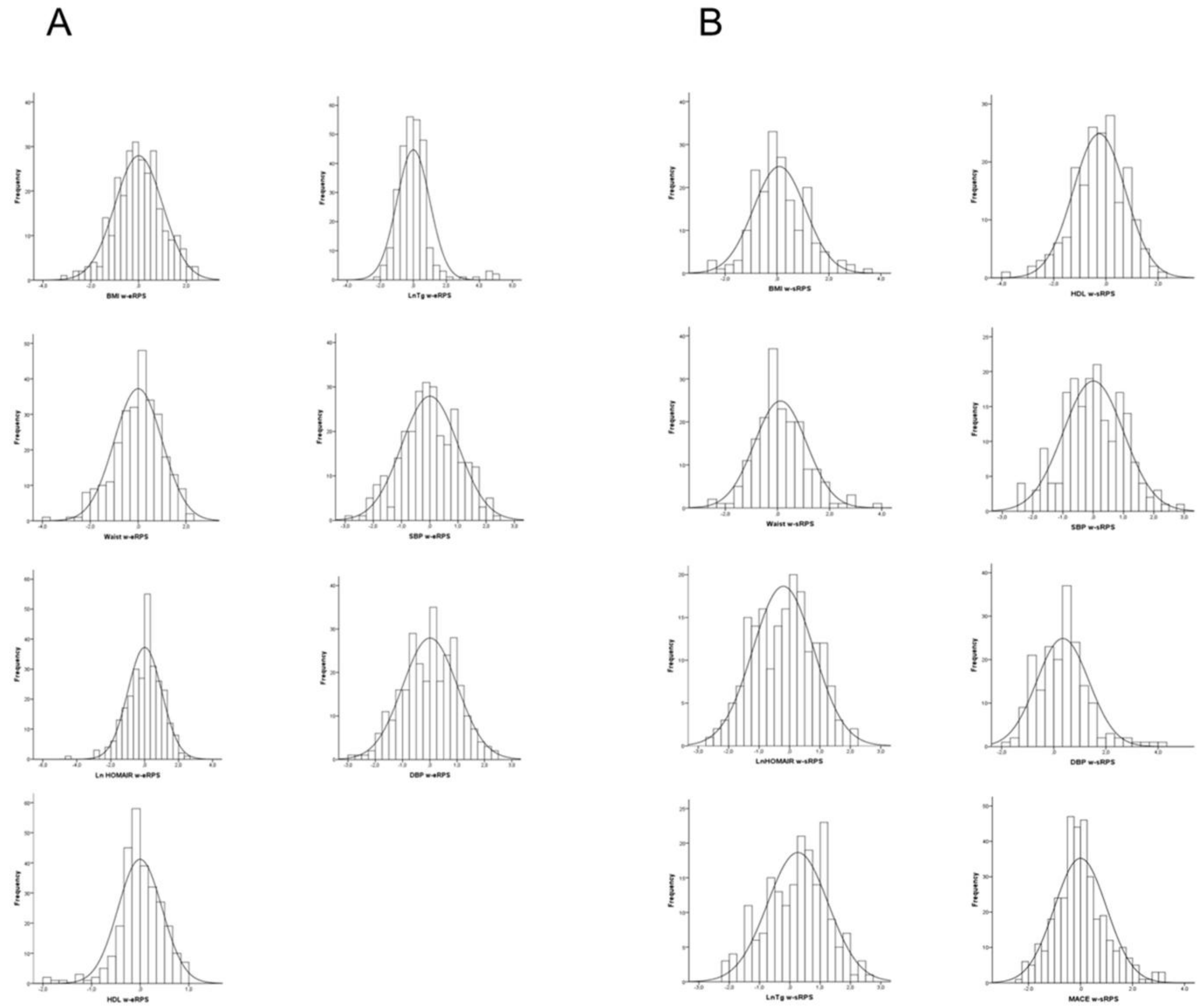
A
B
Frequency
BMI w-eRPS
LnTg w-eRPS
Waist w-eRPS
SBP w-eRPS
Ln HOMAIR w-eRPS
DBP w-eRPS
HDL w-eRPS
BMI w-sRPS
HDL w-sRPS
Waist w-sRPS
SBP w-sRPS
LnHOMAIR w-sRPS
DBP w-sRPS
LnTg w-sRPS
MACE w-sRPS

Figure S2

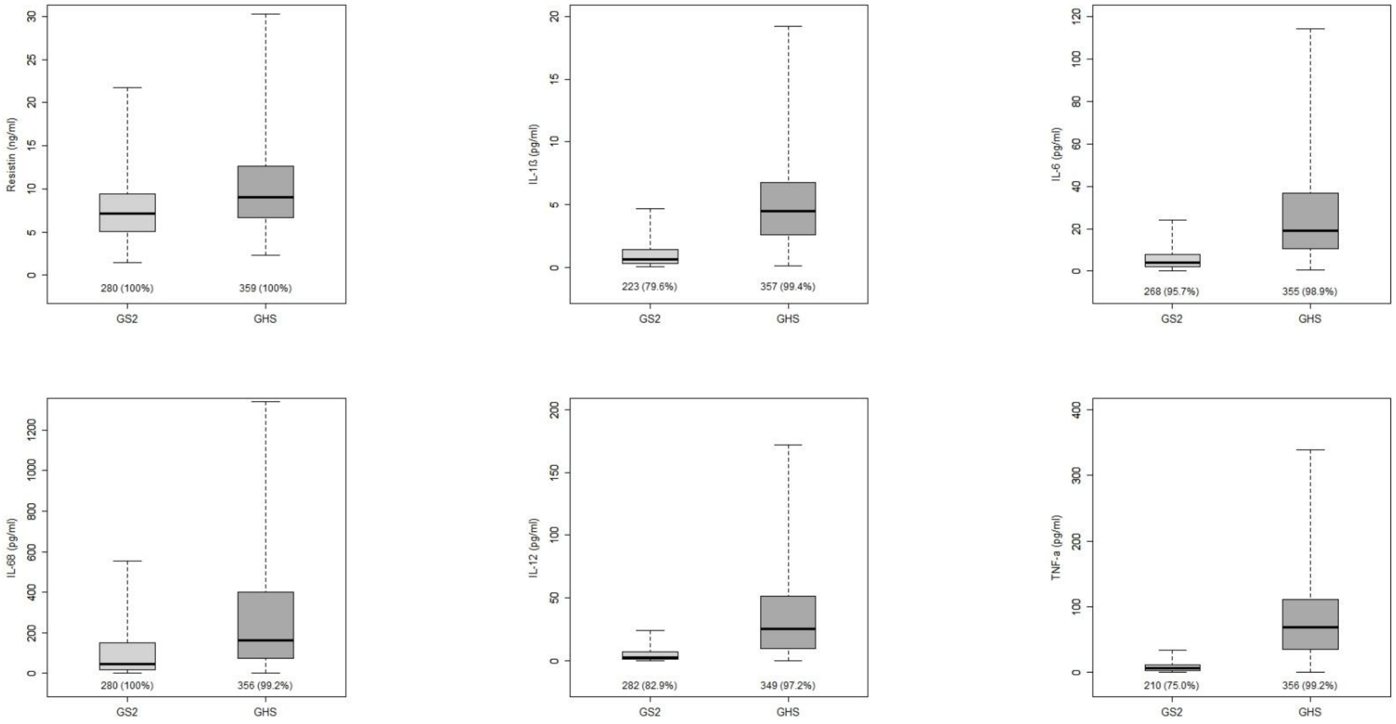

Resistin (ng/ml)
280 (100%)
359 (100%)
GS2
GHS
IL-1ß (pg/ml)
223 (79.6%)
357 (99.4%)
GS2
GHS
IL-6 (pg/ml)
268 (95.7%)
355 (98.9%)
GS2
GHS
IL-68 (pg/ml)
280 (100%)
356 (99.2%)
GS2
GHS
IL-12 (pg/ml)
282 (82.9%)
349 (97.2%)
GS2
GHS
TNF-a (pg/ml)
210 (75.0%)
356 (99.2%)
GS2
GHS
